# Serum Metabolomics of Slow vs. Rapid Motor Progression Parkinson’s Disease: a Pilot Study 

**DOI:** 10.1371/journal.pone.0077629

**Published:** 2013-10-22

**Authors:** James R. Roede, Karan Uppal, Youngja Park, Kichun Lee, Vilinh Tran, Douglas Walker, Frederick H. Strobel, Shannon L. Rhodes, Beate Ritz, Dean P. Jones

**Affiliations:** 1 Clinical Biomarkers Laboratory, Emory University, Atlanta, Georgia, United States of America; 2 Division of Pulmonary, Allergy and Critical Care Medicine, Emory University, Atlanta, Georgia, United States of America; 3 Department of Chemistry, Emory University, Atlanta, Georgia, United States of America; 4 Department of Civil and Environmental Engineering, Tufts University, Medford, Massachusetts, United States of America; 5 Department of Epidemiology, University of California Los Angeles School of Public Health, Los Angeles, California, United States of America; 6 School of Biology, Georgia Institute of Technology, Atlanta, Georgia, United States of America; 7 College of Pharmacy, Korea University, Sejong City, Korea; National Institutes of Health, United States of America

## Abstract

Progression of Parkinson’s disease (PD) is highly variable, indicating that differences between slow and rapid progression forms could provide valuable information for improved early detection and management. Unfortunately, this represents a complex problem due to the heterogeneous nature of humans in regards to demographic characteristics, genetics, diet, environmental exposures and health behaviors. In this pilot study, we employed high resolution mass spectrometry-based metabolic profiling to investigate the metabolic signatures of slow versus rapidly progressing PD present in human serum. Archival serum samples from PD patients obtained within 3 years of disease onset were analyzed via dual chromatography-high resolution mass spectrometry, with data extraction by xMSanalyzer and used to predict rapid or slow motor progression of these patients during follow-up. Statistical analyses, such as false discovery rate analysis and partial least squares discriminant analysis, yielded a list of statistically significant metabolic features and further investigation revealed potential biomarkers. In particular, N8-acetyl spermidine was found to be significantly elevated in the rapid progressors compared to both control subjects and slow progressors. Our exploratory data indicate that a fast motor progression disease phenotype can be distinguished early in disease using high resolution mass spectrometry-based metabolic profiling and that altered polyamine metabolism may be a predictive marker of rapidly progressing PD.

## Introduction

Parkinson’s disease (PD) is a complex, multisystem disorder of unknown etiology that presents a broad array of symptoms and pathological features affecting organs throughout the body [1]. PD is commonly described as a progressive neurodegenerative condition caused by the preferential loss of dopaminergic neurons present in the substantia nigra pars compacta; however, other brain regions, like the locus coeruleus, are also affected [2]. Motor symptoms include bradykinesia, rigidity and postural instability. Depression, constipation, loss of the sense of smell and sleep disturbances are included in the spectrum of non-motor symptoms reported by PD patients [3].

The heterogeneity of PD symptoms suggests that different disease subgroups exist and that these subgroups may possess distinct etiological processes [4]. Due to this heterogeneous nature, the quest for reliable biomarkers that can predict disease onset, progression and/or outcome is ongoing. To date, the most well defined PD biomarkers involve neuroimaging techniques that determine the extent of nigrostriatal degeneration [5]. Biochemical biomarkers that reflect PD pathogenesis are greatly needed due to the fact that degeneration of the dopamine producing neurons is an irreversible process; therefore, biomarkers may aid in early detection and more effective disease management. These biomarkers need to be detectible in accessible samples, such as blood, saliva and cerebral spinal fluid [5]. In an effort to discover viable biomarkers, researchers have begun to employ ‘omics’ approaches in combination with bioinformatics and biostatistical methods to aid in the discovery of these very important biomarkers present in complex biological samples.

The term ‘metabolomics’ can be defined as the study of global profiles of all metabolites in a given sample [6]. These metabolites can include endogenous intermediary metabolites, pharmaceutical metabolites, environmental chemicals, and chemicals arising from gut microflora. Many different platforms can be utilized to study metabolomics. Techniques like proton nuclear magnetic resonance (NMR), magnetic resonance spectroscopy (MRS), high performance liquid chromatography (HPLC) with electrochemical detection, and mass spectrometry (MS) are commonly used. Unfortunately, while metabolomics is the endpoint of the “omics cascade” and is closest to phenotype, there is no single platform that can currently analyze all metabolites [7]. Our “top-down” method of metabolic profiling [8] is aimed at examining the spectrum of metabolites and environmental chemicals present in biological samples. By using high resolution and high mass accuracy mass spectrometry, one can predict the elemental composition to match more than 90% of the most common intermediary metabolites, such as those involved in amino acid metabolism and the TCA cycle [9,10].

The purpose of this pilot study was to utilize high resolution mass spectrometry-based metabolic profiling to identify biomarkers that distinguish between slow and rapid motor symptom progression in PD patients using serum samples collected prior to the observed progression phenotype. Our method detected over 7,700 distinct ions (*m/z*) and further analyses yielded a list of high quality ions, 1,672 *m/z* with a coefficient of variation (%CV) of less than or equal to 10%. In an attempt to discover potential biomarkers that distinguish slower from more rapidly progressing motor symptoms of PD, false discovery rate analyses along with other multivariate statistical approaches were performed, which resulted in lists of potential biomarkers. Finally, it was found that N8-acetylspermidine was increased in rapid progressors compared to either slow progressors or control participants, indicating a potential biomarker associated with rate of motor symptom progression in PD.

## Materials and Methods

This study was approved by the human subjects committee of the University of California, Los Angeles (UCLA). Subjects participated after written informed consent was obtained which followed a discussion of the consent documents with subjects or legal guardians if participants deemed incapable of consenting. 

### Study population

The serum samples analyzed were a subset of participants from a case-control study that enrolled PD patients and population-based controls between 2001 and 2007 in Central California and followed cases until 2011, death or loss to follow-up. Recruitment methods [11], case definition criteria[12], and case follow-up [13] have been described in detail elsewhere. Briefly, of 1,167 PD patients initially invited, 604 did not meet eligibility criteria, 90 were not examined, 104 were examined by our movement disorder specialists but did not meet published criteria for idiopathic PD [14] and 6 provided only incomplete data. We attempted follow-up examinations for the remaining 363 idiopathic PD patients, but at first re-contact 83 (22.9%) patients were deceased, 25 (6.9%) withdrew, 9 (2.5%) could not be found, and 4 (1.1%) were too ill to participate. Altogether, 242 (66.6%) cases were successfully re-contacted but 6 patients could not be evaluated for motor progression and 3 patients were reclassified as not having idiopathic PD. Of 233 idiopathic patients re-examined, 55 were seen once and 178 (76.4%) were seen twice during follow-up. All patients completed interviews for demographic and risk factor data at baseline and during follow-up. 

For the metabolomics analyses presented we selected 80 of 146 Caucasian PD patients with a serum sample and follow-up examinations in the following manner: 23 males and 16 females from among rapid progressors (defined as having an average annual change in UPDRS motor score in the top quartile of the distribution) and 23 males and 18 females from among slow progressors (defined as having an average annual change in UPDRS motor score below the top quartile). We also selected 20 control sera from among 126 sera from Caucasians available from the 341 population controls enrolled (control selection procedures see 11). Prior to sample collection, patients were not required to fast, but were required to abstain from taking their PD medications the morning of the exam. Exams and sample collections were conducted typically between 8 am and 12 pm. Blood samples were collected in red top tubes and were filled completely. The blood was allowed to clot for at least 30 min, but no longer than 60 min. The samples were then centrifuged at maximum speed (2200-2500 RPM) for 15 min. The tube stopper was then carefully removed and the serum was pipetted into a transport vial. These samples were kept on ice during transit to UCLA and upon receipt were transferred into a -80°C freezer. These samples were stored at -80°C until they were shipped to Emory University on dry ice. We attempted to match the three groups of subjects (slow and rapid progressors and controls) as best possible by age (+/- 5 years), gender (male, female), smoking status (never, former, current) and ambient pesticide exposure based on a previously published geographic information system-based computer model [11] (<8, >=8 different pesticides). 

### Assessment of PD Motor Symptom Progression

Study movement disorder specialists performed Unified Parkinson’s Disease Rating Scale (UPDRS) [15] exams at clinics or residences if the patient was unable to travel. Patients were examined off PD medications (i.e. overnight medication withdrawal prior to exam) whenever possible (82% of patients were off medication for baseline exam, 80% for follow-up exams). A list of medications taken by study participants can be found in Table S1. When we were unable to conduct an off exam, we used the on exam to estimate the off exam score for a patient by adding to the patient’s on exam score the difference of the whole study population’s mean off- and mean on-scores. At each exam 6% of patients were unable to perform some motor UPDRS items (e.g. patient was unable to walk due to a missing lower limb); in these cases we either assumed no change from baseline to follow-up for patients with available baseline data or used the population mean for patients with neither baseline nor follow-up values for an item, a conservative approach that may bias our estimates towards the null. 

Annual rate of change in UPDRS motor score was calculated as the difference of the last follow-up and the baseline motor scores divided by the interval of time (in years) between exams. Subjects were classified as rapid progressors if their average annual change in motor score was in the top quartile of the distribution of scores. In the full study population, the mean annual change in motor UPDRS score was 2.53 (sd=2.65; range 0-24.6); 59 subjects were classified as rapid progressors (mean change = 5.89, sd=2.90, range 3.85-24.6) and 174 were classified as slow progressors (mean change = 1.39, sd=1.20, range 0-3.83). In the 80 subjects contributing to this analysis, the mean annual change in motor UPDRS score was 3.65 (sd=3.42, range 0-24.6); 39 subjects were classified as rapid progressors (mean change=5.95, sd=3.48, range 3.85-24.6) and 41 subjects were classified as slow progressors (mean change=1.45, 1.20, range 0-3.57).

### High Resolution Mass Spectrometry-based Metabolic Profiling

To prepare samples for mass spectral analyses, 50 μL of serum was added to 100 μL of acetonitrile and 2.5 μL of a mixture of 14 stable isotope standards. After mixing and incubation at 4°C for 30 min, precipitated proteins were pelleted via centrifugation for 10 min at max RPM on a microcentrifuge at 4°C. Supernatants were transferred to autosampler vials and analyzed using an autosampler at 4°C. Samples were analyzed in triplicate by liquid chromatography-Fourier transform mass spectrometry (Accela- LTQ Velos Orbitrap; *m/z* range from 85-850) with 10 μl injection volume using a dual chromatography setup (anion exchange and C18) and a formic acid/acetonitrile gradient as described by[8]. Electrospray ionization was used in the positive ion mode. Data were extracted using xMSanalyzer [16] as *m/z* features, where an *m/z* feature is defined by *m/z* (mass-to-charge ratio), RT (retention time) and ion intensity (integrated ion intensity for the peak). Putative identification of metabolites was made using the Madison Metabolomics Consortium Database (MMCD) [17] and Metlin Mass Spectrometry Database [18]. Metabolite identities were confirmed via tandem mass spectrometry (MS/MS) and matching fragmentation patterns to those of known standards when possible. 

### Quantification of N8-acetyl spermidine

Quantification of N8-Acetylspermidine (N8-ASP) within the samples was accomplished via a single concentration response factor determined from the average intensity of the N8-ASP protonated adduct [M+H]^+^ within NIST SRM 1950, which was included during analysis of the samples, and its corresponding N8-ASP concentration. The level within each sample was then determined by multiplying the reference standard response factor (2.13 x 10^-5^) by the average intensity determined from the technical duplicates for each individual sample. This technique enabled the absolute quantification of N8-ASP within each sample without sample cohort re-analysis, and only required determination of n8-ASP within the pooled reference sample. The N8-ASP concentration within NIST SRM 1950 was quantified by reverse-phase LC with detection via a high resolution Orbitrap mass spectrometer (Q-Exactive, Thermo Scientific, San Diego CA) operated in both full scan and selective ion monitoring mode (SIM) following positive electrospray ionization. Sample preparation, ionization parameters, C-18 column and gradient method were the same as described previously. The Orbitrap mass analyzer was operated at a resolution of 35,000, with the AGC, injection time and isolation window set to 10^5^ ions, 80 ms and 1 m/z, respectively. Quantification was based on the protonated adduct (188.1757 m/z) +/- 10 ppm mass accuracy, and retention time was confirmed via N8-ASP reference standards ( ≥ 98%, Sigma Aldrich, St. Louis MO) and MS/MS. Determination of N8-ASP concentration in the NIST reference plasma was accomplished by external calibration via a blank corrected response factor calculated from a matrix matched standard containing 5 nM N8-ASP plus the endogenous level. Based on triplicate analysis of the NIST SRM 1950, the 5 nM reference standard and blank standard matrix, the concentration of N8-ASP within NIST SRM 1950 was determined to be 10.1 ± 0.3 nM.

### Metabolomics Data analysis

Bioinformatics and biostatistical analyses included two-sample t-test followed by false discovery rate (FDR) correction, principal component analysis (PCA) and orthogonal signal correction-partial least squares discriminant analysis (OPLS-DA) with principal component loading statistics (PCLS). A two-sample t-test followed by multiple hypothesis test correction using the Benjamini and Hochberg false discovery rate (FDR) method was used to determine metabolites that differed between the groups [19]. We use raw *p* values in a Manhattan plot (-log_10_ *p* vs metabolic feature) to visualize the calculated significance for individual metabolites according to the progression of parkinsonism and identify the FDR at *q*=0.2 with a horizontal line. 

OPLS-DA is a multivariate supervised analysis to display and identify differences between groups. The analysis produces a score plot showing the separation of the groups based on the content of the loading discriminatory metabolites. After autoscaling the data, an orthogonal signal correction removes variation not correlated to classification. PCLS was performed in conjunction with OPLS-DA to identify metabolites that contribute in a groupwise manner to discriminate samples. PCLS uses statistical principles to select the top 5% of metabolites accounting for 95% group identity. 

Topological comparison of the metabolic networks in slow PD and rapid PD conditions was performed using the Weighted Gene co-expression Network Analysis (WGCNA) package in R [19–21]. WGCNA uses hierarchical clustering coupled with topological overlap dissimilarity measures to detect biologically meaningful modules [20]. In network analysis, a module is defined as a subset of nodes that form a sub-network within a network, and these modules are likely to correspond to biological processes and pathways. Spectral data from the slow PD condition samples was used as reference for defining the metabolic modules. These modules were then mapped to the rapid PD samples. The first principal component of each module, eigengene, is used to summarize the ion intensity/expression pattern of the metabolites in that module. The modulePreservation function implemented in the WGCNA R package was used to assess the preservation of modules between slow PD and rapid PD.

## Results

### Subject characteristics

The subjects in this pilot study were from a cohort of PD patients and healthy controls living in the Central Valley region of California. Demographic and phenotypic characteristics of the included subset are described in Table 1. Because of the matching performed in the design of this study, we observe no statistical differences between controls, slow or fast progressors of the pilot subset for these characteristics. The samples selected were not statistically different from the complete study population of longitudinally followed PD patients (Table S2) on distribution of sex, age, education, smoking status, or family history of PD. 

**Table 1:** Study population characteristics.

**Characteristic**	**Control** [n = 20] [no. (%)]	**Slow** [n = 41] [no. (%)]	**Rapid** [n = 39] [no. (%)]
Sex			
Male	10 (50.0)	23 (56.1)	23 (59.0)
Female	10 (50.0)	18 (43.9)	16 (41.0)
Age (years)^a^			
< 60	3 (15.0)	6 (14.6)	5 (12.8)
≥ 60	17 (85.0)	35 (85.4)	34 (87.2)
Average	69.7	68.7	68.9
Range	47 - 84	50 - 87	46 - 83
Education (years)			
0 to < 12	1 (5.0)	3 (7.3)	3 (7.7)
= 12	3 (15.0)	10 (24.4)	15 (38.5)
> 12	16 (80.0)	28 (68.3)	21 (53.8)
Smoking status			
Never smoker	10 (50.0)	26 (63.4)	19 (48.7)
Former smoker	10 (50.0)	15 (36.6)	19 (48.7)
Current smoker	0 (0.0)	0 (0.0)	1 (2.6)
Family history of PD^b^			
None	17 (85.0)	34 (82.9)	35 (89.7)
1 or more members	3 (15.0)	7 (17.1)	4 (10.3)

Note: all subjects are non-hispanic, Caucasians

^a^ Age at diagnosis for PD cases and age at interview for controls

^b^ Self-reported first-degree relative with PD

PD patients were divided into two motor progression subgroups determined by the average annual increase in UPDRS motor score. A five-point per year change in the motor UPDRS has been reported for early, untreated PD patients in placebo arms of clinical trials [22] and is considered a clinically relevant change when assessing improvement due to treatment [23]; our “rapid” progressors on average experienced an annual rate in motor symptom decline of 5.95 points (range 3.85 - 25.6), while the slow progressors mean change in symptoms was only 1.45 points (range 0 - 3.57).

 We have previously demonstrated that subjects lost to follow-up due to death were older and had a higher baseline UPDRS score than subjects followed and that subjects lost to follow-up due to other reason were no different on risk factors from slow progressors [13] 

### Comparisons of PD patients and controls

Extraction of mass spectral data resulted in a list of 11,433 *m/z*, where 7,718 *m/z* were detected in 100% of the sample runs. To determine whether a statistically significant metabolic difference existed between control and PD patients, the 7,718 *m/z* that were detected in all samples were analyzed by false discovery rate analysis (FDR). FDR analysis resulted in a list of 259 *m/z* that were significantly different between controls and PD cases (Table S3). It should be noted that only 17% of these m/z had a match (M+H, M+Na) in the Metlin database. The ions co-eluted with Na^+^ and K^+^ ions and searches in the Metlin database also showed matches to predicted high-resolution *m/z* of Na^+^ and K^+^ forms of several phytochemicals. Because of the large number of possible isomeric structures and a lack of available standards, these were not pursued. Additionally, 15 m/z matched to therapeutic drugs and nutritional supplements commonly used by PD patients. Because of their wide use, there was no way to evaluate the contributions of these compounds to alterations in the metabolic profile. Principal component analysis (PCA), an unsupervised, multivariate statistical procedure, was conducted to determine whether metabolic differences between controls and PD patients could be detected; however, this technique did not result in separation of the two groups (data not shown). 

**Figure 1:**
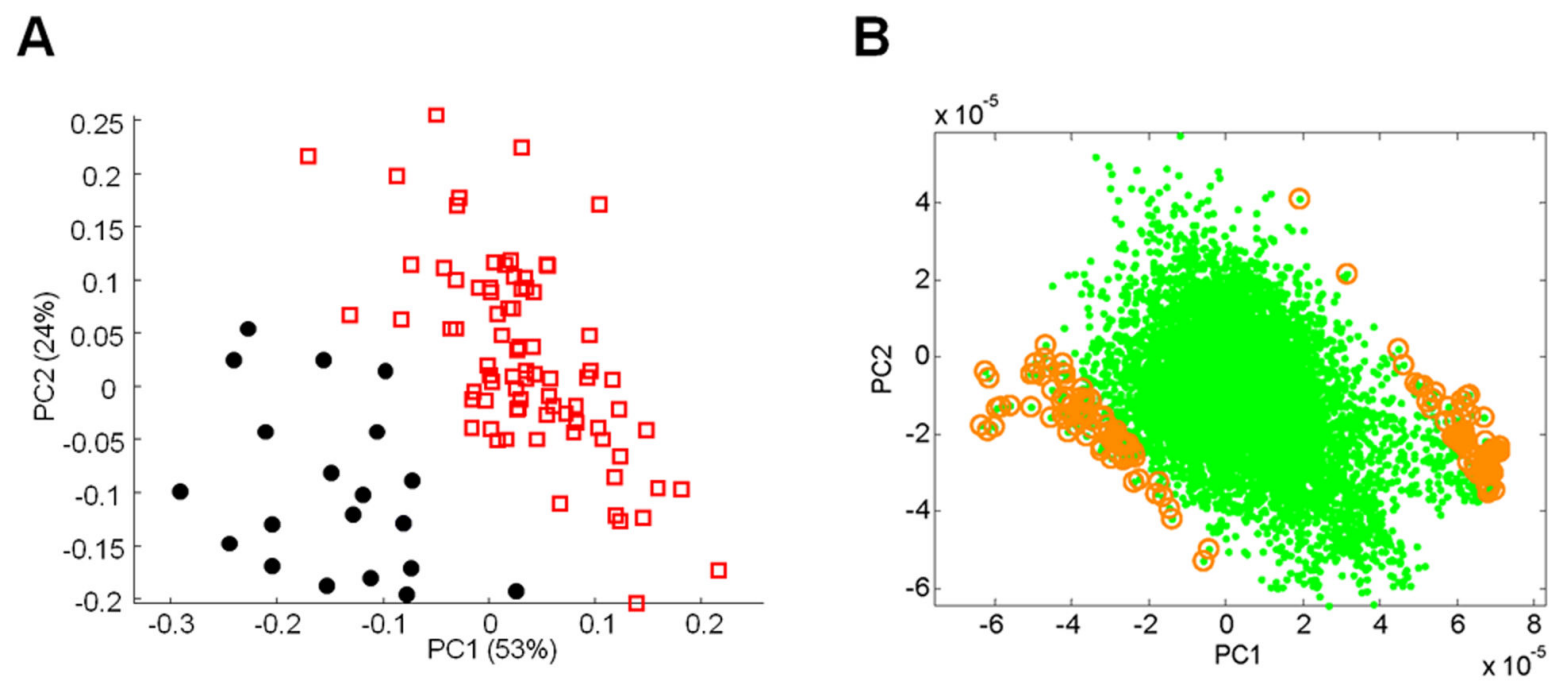
Separation of control and PD using OPLS-DA. OPLS-DA results comparing metabolites from control (black, closed circles) and PD patients (red, open squares) (A). Discriminatory analysis of control and PD patient metabolic data (B). The green dots represent metabolites with 95% correlation to the first two principal components. The gold circles denote the top 1% of these metabolites (154 *m/z*) that most closely associate with control or PD.

A supervised, multivariate technique, orthogonal partial least squares-discriminant analysis (OPLS-DA), was also used to analyze the data. Figure 1A clearly shows that the data from control and PD patients can be separated into two distinct groups, thereby classifying the data from 100 subjects according to disease status. We also employed an additional multivariate statistical approach termed principal component loading statistics (PCLS) to select metabolites that contributed to this group behavior observed using OPLS-DA. This approach provides a complement to FDR and it utilizes loading vectors from OPLS-DA to directly link to a correlation analysis for group separation (Lee et al, manuscript in preparation). 154 *m/z* were found by PCLS to distinguish controls from PD patients (Figure 1B). This list was annotated using the Metlin database (Table S4) and it was found that 76% of these metabolites (117 of 154) were unknowns, meaning that they did not have an M+H or M+Na match in the database. Of the remaining 37 metabolites, some putative identifications of interest included the flame retardant PBDE-99, various tripeptides, phytochemicals and potential drug metabolites. Because of our interest in elucidating specific phenotypic differences between the metabolic profiles of slow progressors and rapid progressors, we chose to further evaluate these two disease subtypes and not compare them to the small group of healthy controls. 

### Rapid versus slow progression

The PD patients were classified into slow- and rapid-progressing phenotypes according to annual increase in UPDRS motor score. 

**Figure 2:**
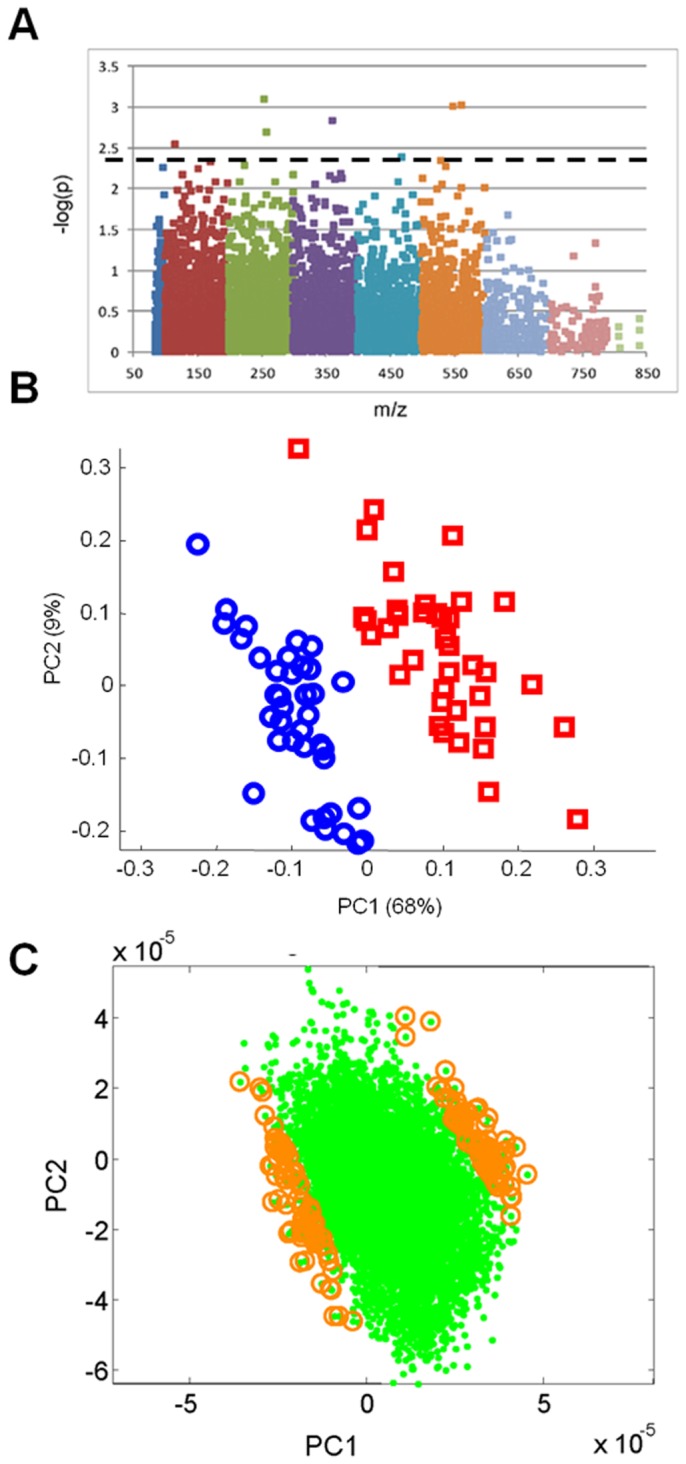
Significant metabolites and separation of slow and rapid PD phenotypes. A manhattan plot (A) shows a number of metabolites that break the threshold of significance (dotted line) when comparing slow vs. rapid PD progression. OPLS-DA results comparing metabolites from slow (blue) and rapid (red) progressors (B). Discriminatory analysis of control, slow and rapid progressor metabolic data (C). The green dots represent metabolites with 95% correlation to the first two principal components. The gold circles denote the top 1% of these metabolites (152 *m/z*) that most closely associate with slow or rapidly progressing PD.

Analysis of global changes in metabolic profiles between the slow and rapid progressors yielded a list of 35 *m/z* that were found to differ between the two groups. A Manhattan plot of all *m/z* with a %CV of less than or equal to 10% (1,672 *m/z*) displays the number of metabolites above the threshold of significance (q=0.2)(Figure 2A). Putative identification of these metabolites can be found in Table S5. 49% of these metabolites did not have a database match (M+H or M+Na) and all of the significant metabolites exhibited higher ion intensity in the rapid progressors compared to slow progressors. OPLS-DA was also conducted and resulted in a separation of slow and rapid progressors (Figure 2B). PCLS showed that 152 *m/z* contributed to the separation between slow and rapidly progressing PD (Figure 2C). Of these 152 m/z, 81 (53%) did not possess a match to common adducts (M+H or M+Na) in the Metlin database (Table S6). Those m/z that did have a database match were putatively identified and these matches included, acetylspermidine, 3,4-dihydroxyphenyl acetaldehyde (DOPAL) and various amino acid metabolites, lipids, phytochemicals, tripeptides and potential pharmaceutical metabolites. 

**Figure 3:**
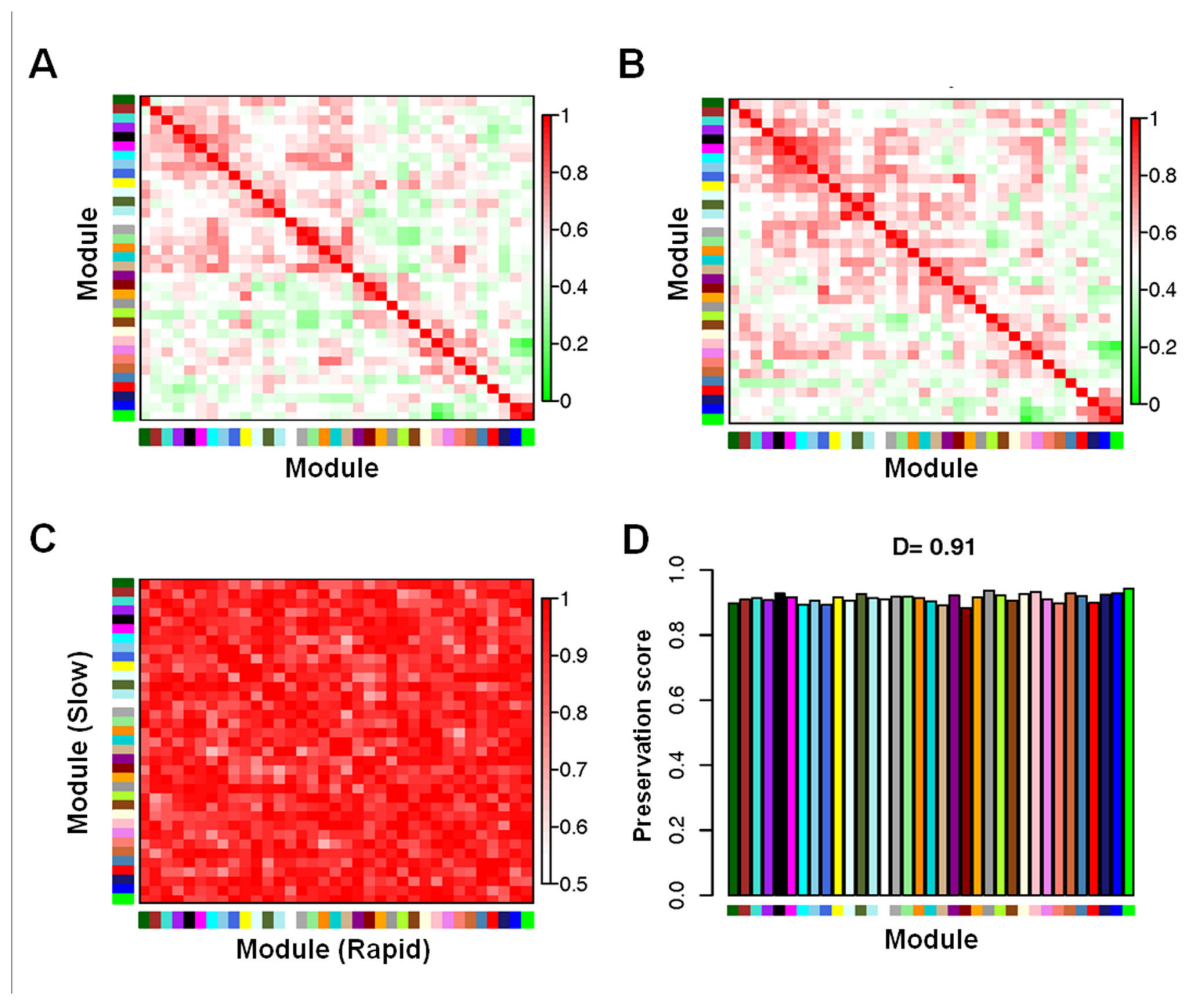
Preservation matrix reveals subtle metabolic differences in slow vs. **rapid PD**. Summary plot of consensus eigengene networks and their differential analysis comparing slow versus rapid progression PD. The eigengene networks in the two sets, slow (A) and rapid (B) are shown as heatmaps. In the heatmaps, red denotes high adjacency (positive correlation) and green denotes low adjacency (negative correlation). The Preservation heatmap (C) shows the preservation network, defined as one minus the absolute difference of the eigengene networks in the two data sets. The barplot (D) shows the mean preservation of adjacency for each of the eigengenes to all other eigengenes; in other words, the barplot depicts the column means of the preservation heatmap.

Topological differences between slow and rapid conditions were evaluated using the module preservation function of WGCNA as described in Methods (Figure 3). 52 modules of metabolites were identified using the slow PD data as a reference set. The preservation of these modules was then evaluated using the rapid PD data as the test set. Heatmaps of the cross-correlation of the modules in each condition, slow PD and rapid PD, are shown in Figure 3A and 3B, respectively. Most of the network connections between the two conditions were preserved although there were few differences as shown in the heatmap of pairwise preservation scores and the bar plots of the preservation score per module, Figure 3C and 3D, respectively. 

**Figure 4:**
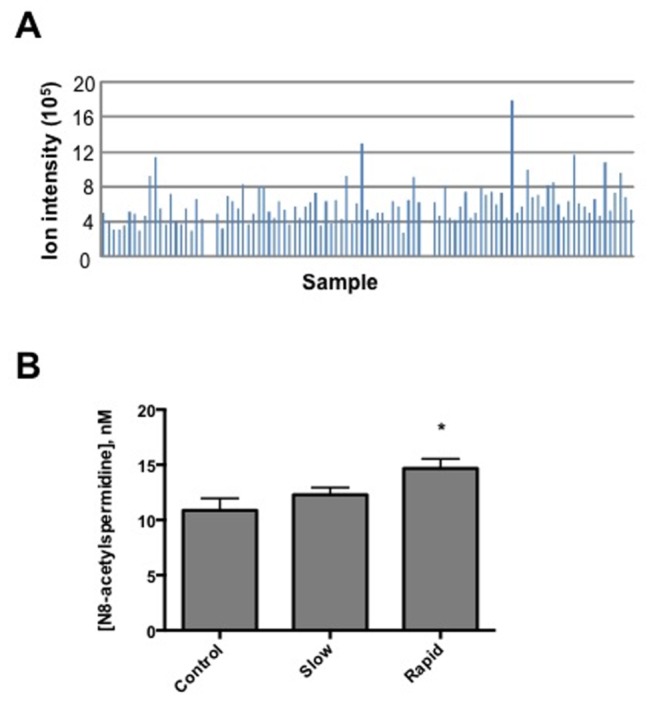
Distinguishing metabolite, N8-acetylspermidine, is increased in rapid PD. Ion intensity results for the metabolite with *m/z* of 188.175 (A). Quantification of serum N8-acetylspermidine (*m/z* 188.175) for control, slow and rapid PD (B). The mean concentration of this metabolite was found to differ significantly between Control and rapid progressors (one-way ANOVA, Tukey post test, *P<0.0.5).

The results from FDR, PCLS, and module preservation analysis suggest that the differences between the two sub-conditions of PD are likely to occur because of small perturbations in the metabolic network structure caused by differential expression of few key metabolites. Of the 12 *m/z* that were common between the FDR and PCLS analyses (Figure S1), *m/z* 188.175 was selected for further study because it was matched (Metlin and MMCD) to an endogenously produced metabolite and was not derived from a pharmaceutical product or phytochemical. Tandem mass spectral analysis of *m/z* 188.175 and investigation of similar mass spectra present in the NIST 11 NIST/EPA/NIH Mass Spectral Library (Scientific Instrument Services, Ringoes, NJ) lead to the conclusion that this metabolite was N8-acetylspermidine. The average ion intensity for N8-acetyl spermidine was plotted for all samples (Figure 4A) and grouped as control, slow and rapid progressors. Utilizing commercially available N8-acetylspermidine as a standard the serum concentrations of this polyamine were quantified (Figure 4B). Rapid progressors had a mean serum concentration of 14.7 nM while the mean concentration in slow progressors and healthy controls was 12.3 nM and 10.9 nM respectively. Because N8-acetylspermidine was found to be significant between slow and rapid progressors via both FDR and PCLS analyses, we also conducted a Pearson correlation analysis to determine what metabolites are highly correlated with this result. Using a correlation of ± 0.3 as a threshold, we generated a list of 163 *m*/*z* (p=0.023) that were highly correlated with N8-acetylspermidine (*m/z* 188.175) (Table S7). This list consisted of 158 m/z that were positively correlated and 5 negatively correlated *m/z*. Of these metabolites, 42% were considered to be unknowns due to the inability to match M+H and M+Na adducts in the Metlin database. Putative identification of the highly correlated metabolites included adrenochrome, 3-O-methyldopa, bilirubin, various lipids, acylcarnitines and tripeptides.

## Discussion

High resolution mass spectrometry-based metabolic profiling and biostatistical approaches can be employed to gain further insight into a disease process and to identify potential biomarkers. The purpose of this pilot investigation was to demonstrate that differences in the serum metabolome of PD patients could distinguish slowly and rapidly progressing forms of PD. The PD patients studied here were similar in distribution of risk factors to the population of PD patients followed longitudinally, and are more likely to be representative of the general population of PD patients that survives 2-5 years beyond their initial physician diagnosis than a clinic-based or clinical trial-based study population. 

About 20% of our study population was not examined in the “off” state. Non- withdrawal, as well as incomplete wash-out of PD medication, is an ever-present consideration even with the overnight medication withdrawal we employed, but longer withdrawal would not be safe or ethical for moderately advanced patients in an observational research setting.  Assuming that PD medications indeed alleviate motor symptoms, both incomplete wash-out as well as non-withdrawal of medication would lower the motor score observed in patients during our exam and possibly misclassify some fast progressors as slow progressors; even so, we detected differences in metabolic profiles between the fast and slow progressors. Additionally, it may be predicted that medications will alter the metabolome; however, this assumes that PD medications’ nature, doses, and dosing schedules influence progression of PD which is not the current consensus. Finally, all of the effects of different medications on the metabolome using a single metabolomic platform could be impossible to detect; therefore, we employed a discovery-based, top-down approach that can easily be adapted to the clinical setting for rapid analysis and diagnosis.

When comparing the serum metabolic profile of controls and PD patients, our results show subtle differences between the PD metabolome and that of healthy controls. The majority of these ions did not have database matches; however, the high-resolution *m/z* values matched several phytochemicals, raising the possibility that unidentified dietary factors could be present that protect against disease development. A previous study utilizing GC-MS to investigate differences in the serum and urine of PD patients compared to controls also reported only a “subtle” metabolic disturbance in the PD patients [24]. The module preservation matrix presented here also indicates small or subtle alterations in the metabolic profile of those with slow or rapid progression PD. Humans are variable in their behaviors and genetic backgrounds so that larger sample sizes and/or sub-classification of populations may be required to clearly define metabolic patterns. Our targeted statistical approach, OPLS-DA, suggests that metabolites can provide a groupwise separation of PD and control using metabolites consistent with known involvement of oxidative stress, mitochondrial dysfunction and perturbed tyrosine metabolism in PD despite being limited to a relatively small number of included subjects. 

Additional metabolomic studies have been conducted in an attempt to identify biomarkers that may distinguish normal controls from PD patients. Two studies investigating metabolic signatures of idiopathic PD [25] and LRRK2-related PD [26] both reported reduced plasma levels of uric acid. Our results did not corroborate this result in that the ion that matched to uric acid in the MMCD and Metlin databases [*m*/*z* 169.0.347 (M+H)] did not differ between PD and controls or between slow and rapid progressors. This result may be due to the differing methods of sample preparation, sample analysis, extraction of mass spectral data and/or characteristics of the study cohort. Future large scale metabolomics studies will attempt to address this discrepancy as well as confirm the results described here.

**Figure 5:**
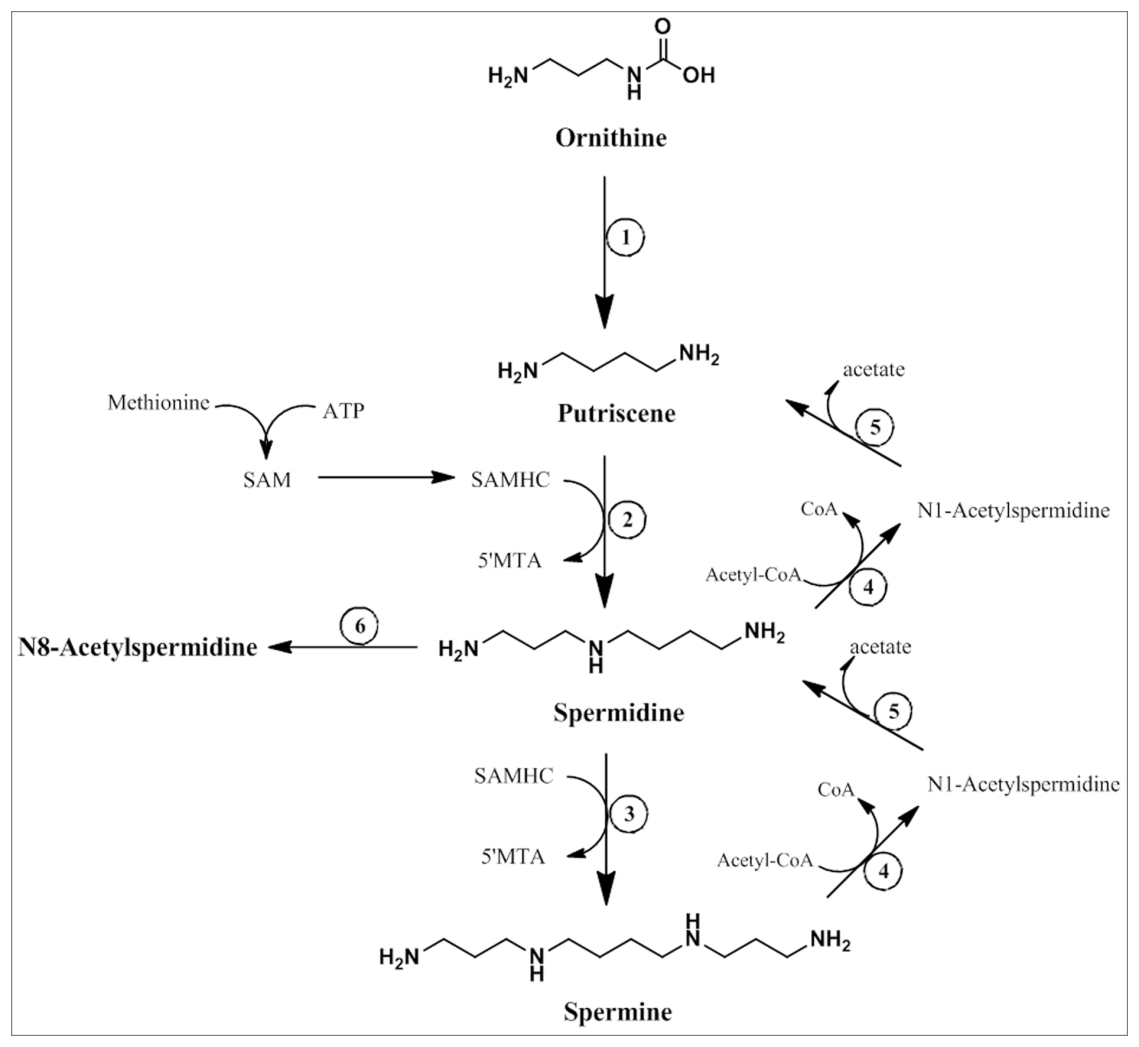
Scehemtic of polyamine metabolism. 1. ornithine decarboxylase; 2. spermidine synthetase; 3. spermine synthetase; 4. spermidine/spermine acetyl transferase; 5. polyamine oxidase; 6. N8-acetylspermidine acetyl transferase. This schematic was adapted from Moinard et al. (2005).

FDR analyses yielded a list of 35 m/z that differed between the two subgroups of PD, slow and rapid progression, and included a polyamine metabolite. N8-acetylspermidine was also found to be one of the significant metabolites that differentiate these two subgroups using PCLS analysis. Polyamines (PA) are an important and ubiquitous group of molecules that includes putrescine, spermidine, and spermine. PA modulate cell growth, proliferation and, due to their positive charge, can interact with DNA and RNA. These interactions can, in turn, alter protein and nucleic acid synthesis, and gene expression [27]. The main sources of PA include dietary intake, cellular synthesis and microbial synthesis in the gut [28]. Figure 5 presents a schematic describing PA metabolism. PA acetylation is an important mechanism by which the cell can modulate PA levels and function [29]. Spermidine contains two sites that can be acetylated, N1 and N8. Acetylation at the N1 position is involved in the interconversion between spermidine and putriscine. N8 acetylation is reported to occur by a nuclear acetyl transferase that is suspected to be involved in epigenomic processes. N8-acetylation of spermidine results in the removal of spermidine from the nucleus and excretion from the cell and eventually the body; an increase in excretion is a mechanism by which the cell can control the intracellular PA concentration [27,30]. It is hypothesized that this nuclear-to-extracellular translocation could be involved in the regulation of cell growth by affecting histone acetylation and having an antiapoptotic effect [30]. Alterations in PA metabolism have been implicated in the mechanism of neuronal degeneration [31] and elevated N8-acetylspermidine levels have been detected in the cerebral spinal fluid samples of PD patients compared to controls suggesting that PA metabolites may provide valid biomarkers for the disease [32]. Another metabolomic study [24] reported an increased level of a “biogenic amine” in the urine of PD patients; however, the identity of this amine was not described. As mentioned previously, N8-acetylspermidine is an excretion product; increases in PA excretion are associated with injury, including traumatic brain injury, as well as neuroinflammation and neuronal cell death [31,33,34]. Additionally, N8-acetylspermidine was shown to increase dopamine production in PC12 cells [35]. The increase in circulating N8-acetylspermidine seen in fast progressing PD cases compared to slower progressing cases early in disease may represent a cellular response to neuroinflammation or may be an attempt to increase dopamine production in existing neurons. 

*In vitro* studies have shown that spermidine can interact with α-synuclein (α-syn), thereby enhancing α-syn misfolding and aggregation [36]. It has also been shown that α-syn genetic variants can predict fast motor symptom progression in patients with idiopathic PD [13]. In addition to the potential effects on neuroinflammation or dopamine metabolism, the enhanced elimination of spermidine in the N8-acetyl form observed in the rapidly progressing patients may represent a protective response exhibited by neurons in an attempt to alleviate or prevent α-syn misfolding and aggregation. This particular mechanism requires confirmation in an *in vivo* model. 

Although speculative, altered PA metabolism may be linked to changes indicating lower lysine and altered acylcarnitine metabolism observed in the PCLS analysis comparing slow and rapid progression and in the list of *m/z* correlated with N8-acetylspermidine. Lysine is a precursor for carnitine, which is required for fatty acid transport into mitochondria for metabolism. Mitochondrial dysfunction has been associated with PD, and several studies of chronic and age-related diseases show altered acylcarnitine metabolism.

Our pilot study demonstrates that high resolution mass spectrometry-based metabolic profiling can distinguish slow progressing PD from rapid and suggests alterations in PA metabolism early in disease may be related to rate of motor symptom progression. Additional studies with larger cohorts will be needed to independently test these concepts. It should be acknowledged that a large percentage of the ions detected corresponded to metabolites that were not present in the current metabolomics databases. Conducting metabolome-wide association studies (MWAS) may also provide additional insight regarding a disease mechanism, even for the metabolites that were not putatively identified [37]. For example, further correlation analyses can reveal previously unknown associations between known and unknown metabolites and metabolic processes. Additionally, metabolic tracer studies are needed to determine whether observed changes in metabolite abundance are related to a metabolic switch in use of PA precursors, enhanced nuclear metabolism of PA or impaired metabolic clearance of PA. Such knowledge could provide a foundation for strategies to identify patients at risk of rapid motor progression and interventions to delay or slow progression in all PD patients.

## Supporting Information

Table S1 **List of Parkinson’s disease medications taken by study participants.**
(XLSX)

Table S2 **Additional demographic characteristics of the study population.**
(XLSX)

Table S3 **m/z found to be statistically significant between control and all PD patients using FDR (q=0.2).**
(XLSX)

Table S4 **m/z found by PCLS to distinguish control from all PD patients.**
(XLSX)

Table S5 **m/z found to be statistically significant between slow and rapid progressors by FDR (q=0.2).**
(XLSX)

Table S6 **m/z found by PCLS to distinguish slow from rapid progressors.**
(XLSX)

Table S7 **m/z highly correlated to N8-acetylspermidine.**
(XLSX)

Figure S1 **Venn diagram displaying the overlap between FDR and PCLS analyses comparing slow and rapid progressors.**
(TIF)
