# Supplementary figures and images for: Serum Metabolomics of Slow vs. Rapid Motor Progression Parkinson’s Disease: a Pilot Study
Source: PLoS One. 2013 Oct 22;8(10):e77629. doi: 10.1371/journal.pone.0077629 (PMC3805572; doi:10.1371/journal.pone.0077629)

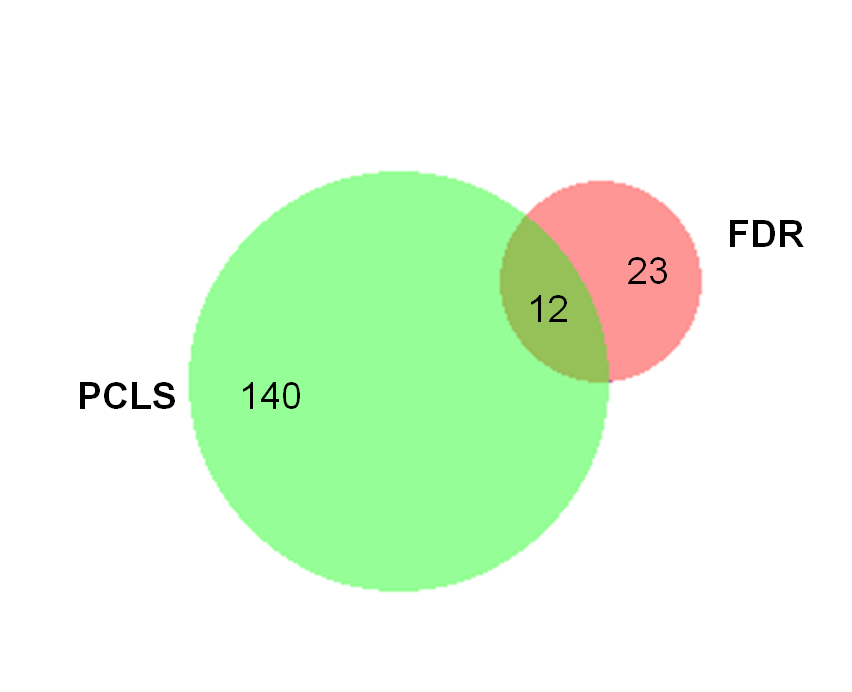

Supplement: Figure S1 — Venn diagram displaying the overlap between FDR and PCLS analyses comparing slow and rapid progressors. (TIF) [file pone.0077629.s008.tif]
